# Response of Coffee Farms to Hurricane Maria: Resistance and Resilience from an Extreme Climatic Event

**DOI:** 10.1038/s41598-019-51416-1

**Published:** 2019-10-30

**Authors:** Ivette Perfecto, Zachary Hajian-Forooshani, Aaron Iverson, Amarilys D. Irizarry, Javier Lugo-Perez, Nicholas Medina, Chatura Vaidya, Alexa White, John Vandermeer

**Affiliations:** 1 School for Environment and Sustainability, University of Michigan, Ann Arbor, MI USA; 2 Department of Ecology and Evolutionary Biology, University of Michigan, Ann Arbor, MI USA; 3 Environmental Studies Department, St. Lawrence University, Canton, NY USA; 4 Departamento de Biología, Universidad de Puerto Rico, Recinto de Utuado, Utuado, Puerto Rico

**Keywords:** Agroecology, Climate-change ecology

## Abstract

Resistance and resilience have become important concepts in the evaluation of disturbance events, providing a framework that is useful in light of the expected increase in frequency and occurrences of hurricanes as a consequence of climate change. Hurricane Maria landed on Puerto Rico as a category 4 storm in September of 2017. Among the affected elements were agricultural systems, including coffee agroecosystems. Historically, coffee has been a major backbone of the island’s agricultural sector. Grown with a range of management styles, the coffee agroecosystem provides an excellent model system to study the resistance/resilience of agroecosystems faced with hurricane disturbance. Sampling 28 farms and comparing pre-hurricane data (2013) with post hurricane data we find that management style had only a small effect on either resistance or resilience, likely due to the especially strong nature of the storm. Rather, the socio-political context of individual farms seems to be a more useful predictor of resilience.

## Introduction

Caribbean islands are highly vulnerable to hurricanes. Recent models of the effects of climate change predict substantial increases in the intensity and the frequency of the most intense tropical cyclones in the Atlantic and increases on the order of 20% in the precipitation rate within 100 kilometers of the storm center^1,2^. While a great deal is known about meteorological and physical aspects of such storms, only recently has the enormous impact on ecological systems become part of the general narrative. Agriculture, in particular, is highly impacted by hurricanes in the region with recent estimates putting export losses due to hurricanes between 18% (for disaggregated data) to 80% (for aggregated data)^3^. Coffee, one of the most important crops in Latin America and the Caribbean, offers an ideal vehicle for the study of resistance and resilience of agroecosystems to hurricane disturbance since it is produced in a variety of management systems^4^. Hurricane Maria, was the strongest hurricane to hit Puerto Rico since 1928. The hurricane made landfall in Puerto Rico as a category 4 hurricane with sustained winds of 155 mph on September 20^th^ 2017^5^. Here we report on how the coffee production style (e.g. the intensification gradient, the investment capital, or the contained agro-biodiversity) contributes, first, to the *resistance* that the farms have against the impact of this category 4 hurricane, and second, to the *resilience* of the farms, less than a year after the landfall of the hurricane.

Coffee production has been categorized along a management intensification gradient that goes from highly diverse shaded systems with a forest-like canopy (i.e. less intensified), to coffee monocultures with no shade trees (i.e. more intensified)^4,6^. In the context of climate change, an important research question that emerges is whether the style of production makes a difference with regard to the damage incurred by hurricanes. Furthermore, of important conservation concern, it is important to understand how this damage affects biodiversity and its recovery.

**Figure 1:**
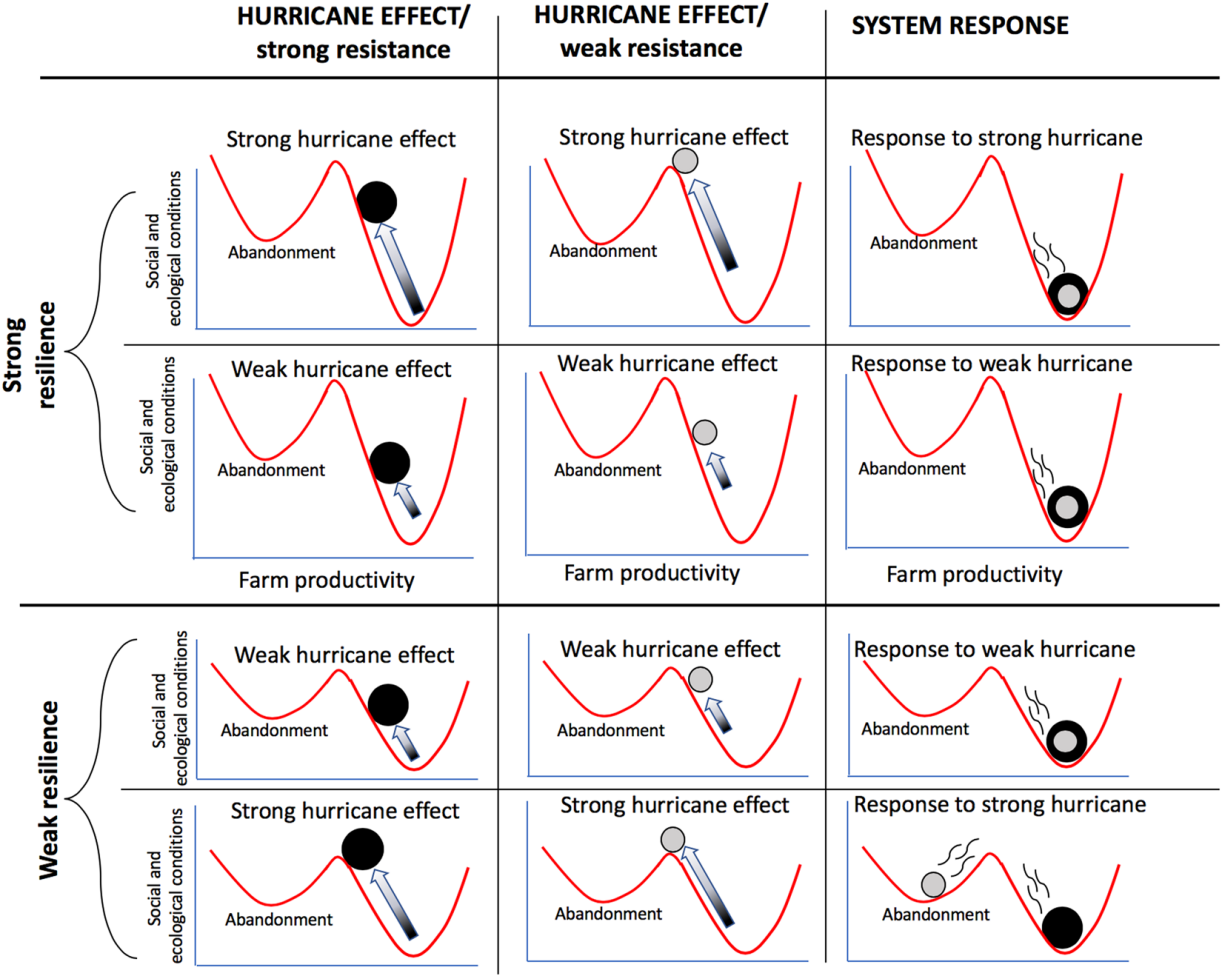
The “ball-in-well” metaphor for resistance and resilience, as applied to hurricane disturbance in the coffee system. The two wells represent alternative production syndromes along the productivity gradient, emerging from the “dynamic background”, which is to say all of the ecological, economic and social forces that determine the farm productivity. This metaphorical representation is commonly employed in the resistance/resilience literature^1^, and is adapted here for the specific application to hurricane disturbance. The actual position of the system is represented by a ball that tends to fall to the bottom of one of the wells. The hurricane is thought of as a force pushing the ball upwards (the shaded arrows represent the force of the hurricane, moving the ball from the dark shaded part of the arrow to the light part of the arrow). A “resistant” system is represented as a larger (heavier) ball which, in consequence, does not respond much to the hurricane. A less resistant system is represented as a smaller (lighter) ball, which responds strongly to the perturbation (i.e. the hurricane). The general expectation is that the hurricane is likely to “encourage” the system to move from a healthy productive system to a low production system and perhaps abandonment. However, if the system has either strong resistance and/or strong resilience, it will go back to the healthy system, while if resistance and resilience are weak, the system will move to a low production system, an alternate state (or “regime” or “syndrome”). The larger heavily shaded ball represents a strongly resistant system while the light shaded ball represents a weakly resistant system.

The concepts of ecological resistance and resilience have been central ideas in ecology for decades^7,8^. In elementary theory, response to disturbance is usually thought of as either stable or unstable, the former, a key framing for the idea of ecological resilience, the latter a metaphor for collapse. This dynamical framing has been a cornerstone of ecological theory since the early 1920s when Lotka and Volterra established the foundational equations for consumer/resource dynamics. Stability in the sense of dynamical systems (technically a negative real part of the system’s eigenvalues) is a well-defined concept, and tying the qualitative idea of resilience to this idea of stability is an easy metaphor. Resistance is somewhat more difficult. Depending on both the strength of the perturbation and the size of the basin of attraction, a resistant system can only be defined with respect to the size of the perturbation, and is effectively the area of the basin of attraction of the stable point (or cycle) in question. Although both ecological resistance and resilience are frequently contextualized together, they are in fact distinct categories^9^. Resistance is the degree to which a system is not affected by a disturbance, while resilience is the degree to which a system, after being perturbed, returns to the original state. The resilience literature usually begins with the ball-in-well metaphor (Fig. 1), a useful framework for explaining the general idea, though even at this level of generality, obvious complexities emerge^10^. Given this theoretical framing, substantial meaning in real-world applications is not always easy, but the underlying meaning of the concepts is evident, and is presented in Fig. 1.

The main factors of hurricanes that might be relevant to resistance and resilience of agriculture are wind, excessive rainfall, and landslides. The relationship between canopy cover and wind is well known^11^, with a general expectation that shade trees will act as wind breaks and damage will be less severe in agroforestry systems than in systems without trees^12–14^. However, such expectations are conditioned on normal wind gusts, not the truly catastrophic winds of a category 4 hurricane. It is not clear whether shade trees would protect the coffee bushes from such strong winds, or whether the trees themselves would be uprooted or snapped and actually damage the coffee. Previous studies on the impact of hurricanes on natural forests in Puerto Rico demonstrate that this is a clear possibility^15^. Excessive rainfall is likely to affect the farming operation in two ways, local puddling and soil erosion^16,17^. Related to the soil erosion effect of rainfall, the damage done by landslides is well-known generally^18,19^, and specifically with regard to coffee farms^20^. A preliminary assessment by the US Geological Survey (USGS: https://landslides.usgs.gov/research/featured/2017/maria-pr/) shows that in the central western region of Puerto Rico, where most coffee farms are located, although most areas had fewer than 25 landsides per Km^2^, there were some areas with more than 25 landslides per Km^2^, while others had no landslides in cells of four Km^2^. Preliminary reconnaissance of the coffee growing region (three months after the hurricane – unpublished observations by JV and IP) suggested that the damage from wind and landslides was severe, but quite heterogeneous, with some farms suffering minimal damage, while others in the general vicinity seemed devastated.

We expect evidence of damage to the agroecosystem on all farms immediately after the hurricane, as has been reported for natural systems^21–28^, and we expect the degree of damage (the resistance) to be correlated with the management system, here reflected in the average percent canopy cover. However, we also expect recuperation of the farms (the resilience) to be less evident in the more intensively managed systems (those that had no buffering from a tree canopy). Overall, we expect the coffee systems with lower intensification (diverse and shaded systems) to have higher levels of resistance and resilience than the more intensified systems with lower levels of diversity, as suggested by the limited literature on this point^20,29,30^.

## Results

### Resistance

**Figure 2:**
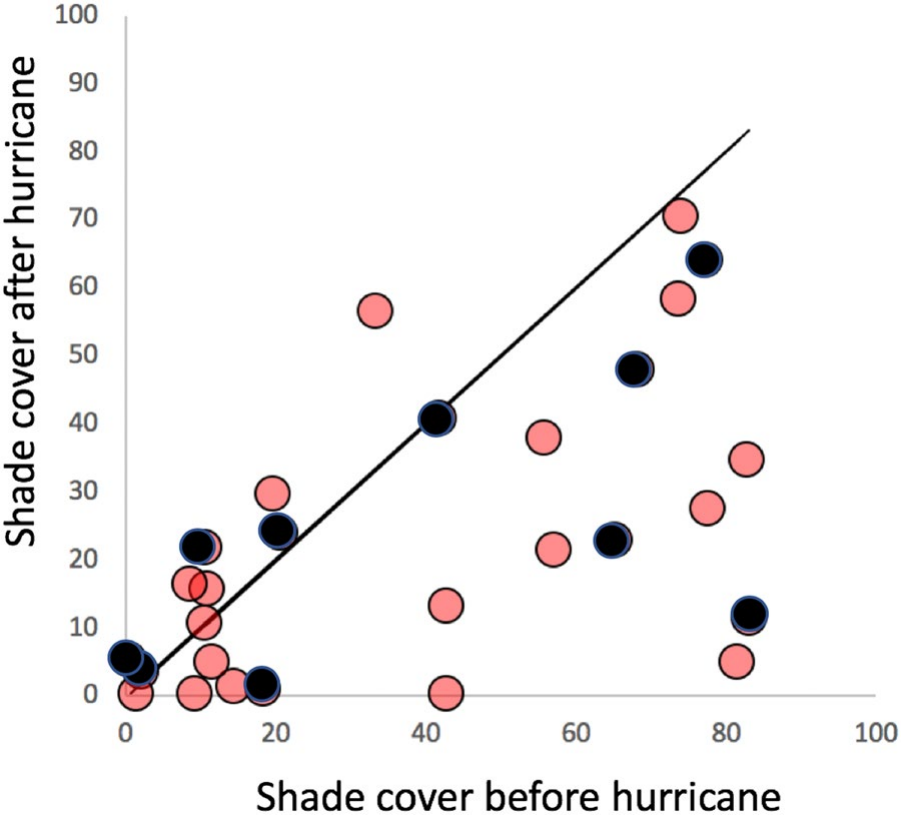
Initial estimate of hurricane damage, comparing estimates from Iverson (2013)^52^ and from the current study (2018). Scatter plot comparison, dark shading representing the 10 farms intensively sampled for pests.

As part of the generalized resilience narrative, resistance refers to the system’s ability to withstand the damaging effects of the disturbance (metaphorically, the mass of the ball in Fig. 1). In the case of the coffee system we expect that the less intensively managed farms (i.e. the ones with more shade trees at the time the hurricane hit Puerto Rico) will exhibit more resistance than the more intensively managed farms (those with less shade trees), largely due to the protective effects of the shade trees (e.g., wind breaks, weed control, fertilization). In Fig. 2 we display the canopy cover for the 28 sampled farms before and after the hurricane. Although the reduction in canopy cover generally is obvious, there is no suggestion that the proportion of canopy cover reduction is somehow related to the level of canopy cover observed on the farm prior to the hurricane (Figs. 2, S1). All that is evident is that the majority of farms lost a great deal of canopy cover, an average of 37.5% canopy loss. The overall canopy loss was from an average canopy cover of about 40% before the hurricane to about 25% after the hurricane. This result is somewhat surprising given the expectation that shade cover, especially in the form of larger trees, is likely to operate as a windbreak^31^. It may be the case that Hurricane Maria was an especially strong hurricane such that vegetative wind breaks were ineffective.

**Figure 3:**
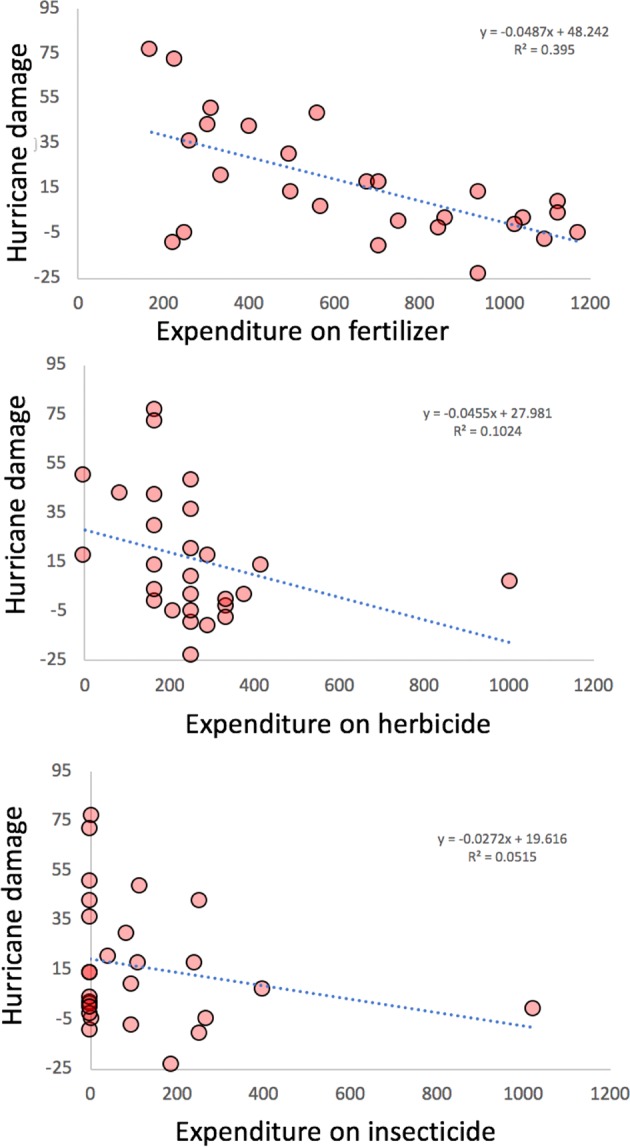
Effects of management intensity on hurricane damage. Intensity figures are from interviews with farmers in 2013.

Management indications for coffee farms are usually associated with canopy cover, or what is frequently referred to as shade cover in coffee farms. Tying such a measure to hurricane damage is obviously not possible since one of the main damages of the hurricane is reduction in canopy cover. It is evident, at a glance (Fig. 2), that the difference between canopy cover before and after the hurricane (the relative residual deviation from the 45-degree line) is not related to the amount of canopy cover before the hurricane. However, from farmer interviews in 2013 we have possible surrogates for management intensity in farmer-reported expenditures for (1) insecticides, (2) herbicides and (3) fertilizer. We take each of those as an indication of management intensity (the more money spent the higher the intensity). Looking at the sum total expenditure on these three inputs as a measure of management intensity, there is a significant regression of hurricane damage (difference between pre- and post-hurricane canopy cover) (R^2^ = 0.326). This relationship suggests that the farms most resistant to hurricane damage were those that were more intensively managed, which is the reverse of what we expected. However, if we disaggregate the three components of intensity, we find that all of that relationship is due to the expenses on fertilizer (organic or conventional) (Fig. 3) (R^2^ = 0.395), negating the tentative conclusion that more intensive management generally leads to resistance. It is thus not possible to make a generalized statement about management intensity, but the relationship between damage and fertilizer is clear and notable.

Interviews with the ten farmers resulted in estimates of damage that were remarkably consistent. All farmers who had shade trees on the farm reporting significant damage to them. All farmers reported a complete loss of the berries that had been maturing at the time of the hurricane, severe damage to the coffee bushes themselves, extensive deposits of debris, and damage to most all other crops. Reports on time passing before the farm was workable (or the farmer was able to do so) varied from “less than a week” (3 farms), to up to three months (5 farms), to more than 6 months (2 farms). Projected harvest for 2018 averaged 0.86 *quintales/cuerda* of green coffee bean (219 kg/ha), whereas the normal for these farms before the hurricane averaged approximately *5 quintales/cuerda* (1272 kg/ha) for the years 2010–2012.

### Resilience

Resilience of the farms is somewhat more difficult to measure than resistance. While it is difficult to judge resilience in less than a year after the hurricane, we can look at some indicators of potential resilience through an analysis of pests and their potential controls. Comparing farm level management to the intensity of pest attacks for six key pest groups (herbaceous vine cover, flatid leafhoppers, green coffee scales, coffee berry borer, coffee leafe miner, and coffee rust disease), some interesting patterns emerge.

**Figure 4:**
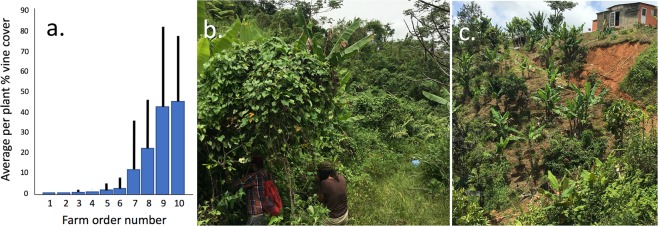
Vine coverage on surveyed farms. (**a**) Average per plant percentage vine cover over ten farms. (**b**) Farm number 10. Note researchers working under the vine coverage to sample insects on the coffee plant, and coffee plants to the rear completely obscured by vine coverage. (**c**) Farm number 2, having been cleared by hand of all weeds before vines could gain traction.

With regard to vine cover, the average percentage vine cover per bush falls into two qualitative categories, almost none and substantial (Fig. 4), reflecting our casual observations of many more farms scattered across the landscape. Of the ten sampled farms, six had low vine coverage (on average less than 10 per cent). Of these, two farms controlled weeds shortly after the hurricane by spraying herbicides (which cannot be done after the vines grow over the coffee plants), two of them controlled the weeds shortly after the hurricane by direct cutting the stems, even though the cost of labor was excessive, and two of them had sufficient canopy cover to begin with and experienced minimal damage, such that vines never became a problem. Labor costs are high and even higher once the vines take over since each tree has to be stripped of the vines, without damaging the coffee tree, a daunting task. In the case of farms that had a high level of canopy cover before the hurricane (e.g., the farm in Fig. 4c), if the shade trees experienced extensive damage (as was the case on farm 2, in Fig. 4c), the felled shade trees had to be cleared first, and then the encroaching weedy vegetation had to be stripped. In the four farms with extensive vine coverage, for one reason or another, the farmer did not have the resources (funds for labor and/or herbicide), to control the vines after the hurricane. This was the case in farm number 10 (Fig. 4b). From both these data and our conversations with farmers and our qualitative observations, the vine cover issue is perhaps the most critical issue in post hurricane resilience, at least with regard to pest problems. In other words, the implicit variable “farm level management” is here reflected in the socio/economic/political ability of the farmer or farm family to engage in effective weed control immediately after the hurricane.

**Figure 5:**
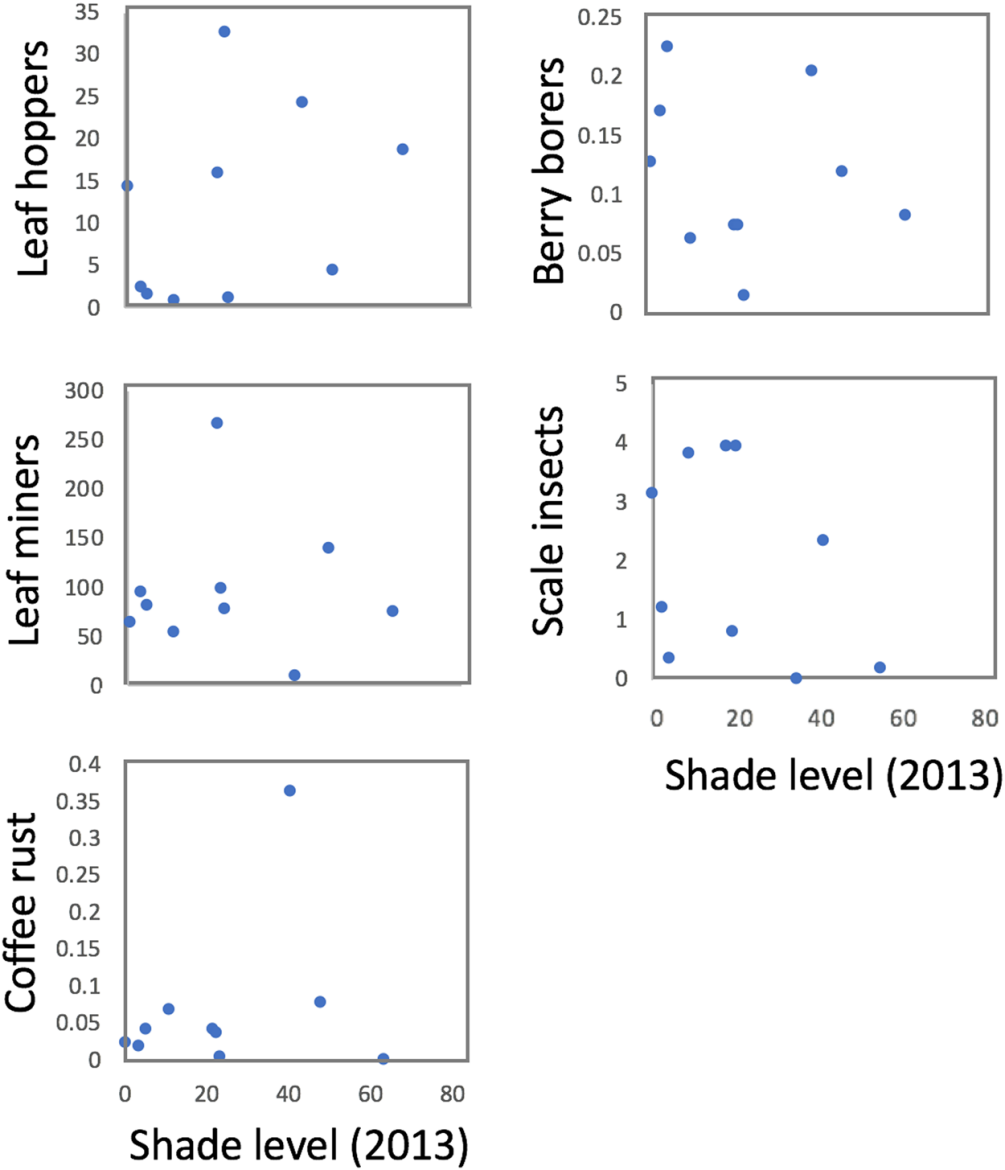
Relationship between five key pest species in post hurricane 2018 and the percent of shade level (canopy cover) (x axis) in 2013.

An alternative measure of farm-level management before the hurricane is the quantity of canopy cover on a farm. Resilience might be encouraged if the response of pests and diseases were correlated with the canopy cover, with a higher percentage of canopy cover resulting in lowered post-hurricane abundance of pests. We find no evidence of any such relationship (Fig. 5). It is worth noting, as is evident in Fig. 5, that much like the vine data discussed previously, there is a tremendous amount of variability among the farms. Of special note are single outliers in the case of the leaf miner and the coffee rust disease, both distinct farms. Furthermore, in the case of scales and flatids, there seems to be a bimodal distribution, some farms with many and some farms with few. These large variations were evident qualitatively when taking the data in the field, yet no obvious corollaries could be conjured to explain them.

**Figure 6:**
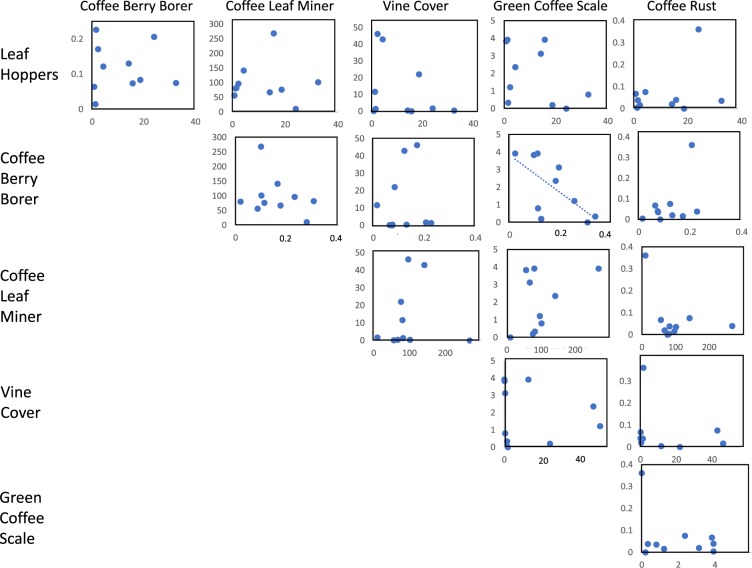
Relationship at the farm level between the six pest species evaluated in this study. Dashed line represents significant lineal relationship at p < 0.05.

If there were some clue to resilience in the pest distribution data, it may be reflected in the correlations among the various pest species, with “non-resilient” farms (with respect to pests) exhibiting generally positive correlations among the various pest problems and resilient farms not showing any pattern. In Fig. 6 we show all the relevant correlations at the farm level for the six pests involved. For the most part there are no correlations among any of the species, suggesting that farm-to-farm variability is effectively stochastic. As mentioned above, no evident clues suggest why one farm should have a large number of some species but not others. In Fig. 6, one particular relationship stands out (the only one for which a statistically significant relationship is evident – Pearson correlation = −0.654, p < 0.03), the negative relationship between the coffee berry borer and the total number of scales. An evident hypothesis for this relationship is the relationship that both have with ants^32^. Ants of various species have mutualistic relationships with the green coffee scale^33–35^, and are predators on the coffee berry borer^32,36–38^. Large numbers of scales may indicate high activity of these ants, which then may be predaceous on the coffee berry borer.

## Discussion

While the damage from Hurricane Maria in September 2017 was substantial, both from casual observation and before and after measurement of canopy cover (Fig. 2), the expected relationship between style of management (i.e. agricultural intensification) and damage caused by the hurricane was not evident. Rather, an enormous amount of variability was encountered. One farm in particular (farm number 3 in Fig. 4a) was densely intercropped with citrus trees. On this farm, the canopies of the citrus trees were kept relatively low and seemed to form a synergistic windbreak with the canopies of the coffee trees and, according to testimony of the farmer, reduced the direct damage from the wind. Furthermore, a large shade tree that was blown over was “intercepted” by the citrus canopy before actually damaging any of the coffee trees below. According to a census of 90 farms in 2018, 37 percent of them include citrus trees interplanted with the coffee (data not shown). However, the density of citrus on this farm was much higher than any other farm that we censused. Also, on this farm it was evident that younger coffee bushes with less well-developed root systems than the older individuals experienced greater damage, again as reported by the farmer. The results discussed above of fertilizer application may be related to this “strength of root” hypothesis, where older coffee plants have more developed root systems.

It is worth emphasizing here that the assumed resilience of a shaded versus unshaded system that we and others have previously argued^4^, is not in any way rejected as a generalization. The fact is that Hurricane Maria was especially strong and our results suggest that what may be substantial resilience (however defined) associated with the shade coffee management system can be effectively cancelled if the disturbance is especially dramatic, as it was in the case of Hurricane Maria, and may very well be more commonly the case as climate change continues its course.

**Figure 7:**
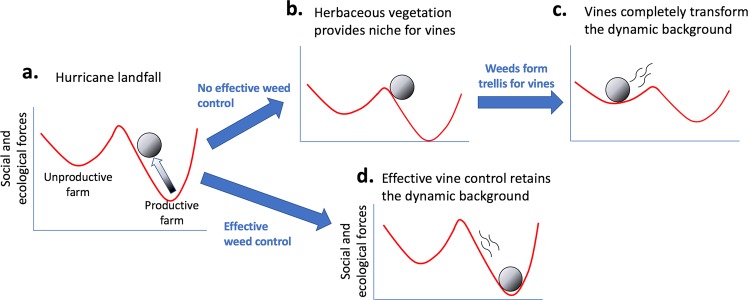
Ball-in-well metaphor as framework for resilience with regard to the essential issue of weed growth (see Fig. 1 for explanation of the framing). (**a**) Landfall of the hurricane causes metaphorical ball to move up in the “productive farm” well, (**b**) resulting from a lack of effective weed control in the immediate post hurricane environment, the ball becomes perched on the border of the two wells (could pushed in either direction, toward the productive farm it originally was or the unproductive farm it could become). (**c**) Vines having transformed the dynamic background (the substance of the wells that attract the balls), the farm becomes transformed into the “unproductive farm” well. (**d**) Contrarily, with effective weed control the ball falls rapidly back to the productive farm well.

While our observations (both quantitative and qualitative) reflect the very large variability in damage and seeming unpredictability of response variables, one pattern seems to emerge clearly, based on the resilience of farms depending on vine management. Judging resilience based on the level of vine coverage (i.e., vines on individual coffee trees, clearly a post hurricane vegetative response) suggests that the combination of management style and direct farmer response combined to set the stage for what we encountered both in our quantitative samples and our casual observations. Farmers able to respond to the hurricane quickly did so, and the current state of their farms seems well positioned for rapid recovery, while those farms whose owners were unable to respond quickly, were put into a distinct ecological regime – excessive vine coverage, and possible loss of production over a long period of time or even abandonment of the farm. Our hypothesis is that the reduction in canopy cover stimulated the growth of grasses, sedges and broadleaf weeds, which facilitated the vines by forming trellises on which the vines were able to quickly reach a substantial height, enabling tendrils to contact coffee bushes. Once on a coffee bush, the vines proliferate rapidly, given the reduction in canopy cover by trees. Thus, the initial response of the farmer to the encroaching weeds dictates which of the two regimes (excessive vine coverage versus healthy farm) will be approached (Fig. 7).

While vines represent a significant “pest” in the system, other more traditional categories of pest have been cited as increasing in importance subsequent to storm and other weather-related events^39–43^. For example, Torres^44^ documented a lepidopteran outbreak in the Luquillo National Forest after hurricane Hugo, and in China, several authors reported the outbreak of the brown plant hopper, a pest of rice, after typhoon Khanun in 2005^45,46^. However, in the case of Hurricane Maria in Puerto Rico, the importance of vines certainly outweighed what seemed to be a rather muted response of other pests in the system.

Data gathered in 2013 on 28 farms provided a point of comparison for data gathered in 2018 on a variety of variables. While canopy cover was unsurprisingly lower after the hurricane, there was no relationship between percent of canopy cover (a measure of management intensity) and damage from the hurricane. In another study in the Caribbean, researchers reported that the amount of basal area prior to a hurricane, determined the effects and recovery of hurricane disturbance^47^. In that study, basal area was reduced in areas with secondary forest and shaded cacao plantations (i.e. high basal area before the hurricane) but remain unchanged in areas with low basal area. Although we did not have basal area measurements before the hurricane, we did indeed have canopy cover measurements before and after the hurricane. This canopy cover data shows no such relationship, perhaps due to the especially intense nature of Hurricane Maria. Using economic data provided by the farmers in 2013, it was possible to show a relationship between management intensity and hurricane damage, but in the opposite direction than was expected. Disaggregating those economic variables, it is evident that the only variable of importance is the amount spent on fertilizer, suggesting a role for strong root structure as a mechanism of resistance. Examining the distribution of various species of pests, we find little evidence one way or another of resilience associated with management.

In addition to the quantitative measurements, there is a general qualitative set of observations that are likely more informative regarding both resistance and resilience. From the farms sampled (10 for major pests and 28 for general damage) and from conversations with those farmers and other researchers seeking to understand the effects of the hurricane, several qualitative observations and tentative conclusions can be offered. First, the size and strength of Hurricane Maria was quite unusual, perhaps negating some management techniques that otherwise would have provided some resistance. For example, canopy cover with relatively large shade trees is likely to be effective at providing some windbreak protection of coffee plants. But when winds are so strong, those trees are toppled, and their trunks and canopies can do considerable damage to the coffee trees below. Also, those farms that happened to be located in deep valleys or with some sort of protection from hills or limestone structures seemed to experience less damage, according to many local accounts in addition to our observations. Finally, the particular path of wind bursts resulted in a lottery effect with some farms experiencing extreme damage and others relatively unscathed. In sum, our casual observations and conversations with many local residents echo our quantitative data that management style has little to do with resistance to the hurricane when the strength of the hurricane is high (Category 4 and 5).

In other Caribbean islands with a stronger traditional agricultural sector, farmers adopt a variety of preventive strategies to minimize the effect of the hurricane on their crops^48–50^. We did not find this to be the case in Puerto Rico after hurricane Maria. However, it is not clear whether preventive measures, such as pruning large trees, could have prevented the toppling of those trees under such strong winds.

Qualitative observations also reflect on the nature of resilience. It is clear that some farms are well on their way to recovery while others are completely devastated with little hope for recuperation at least within a time frame of one to a few years. Here we judge that management did indeed have an important effect, but secondary and complicated. For example, on some farms it was evident that patches with shade trees were not as severely damaged as patches in the open sun. Recuperation of individual coffee plants was dependent to a great extent on how deeply rooted they were, with older coffee plants that had been pruned several times having root stocks that held firm to the ground while younger plants were ripped out of their moorings. Thus, recuperation (resilience) is likely to be far faster for these older more established plants. However, the most evident feature of the resilience narrative was the structure of the weed community. If whatever canopy cover had been on the farm was destroyed (or if the farm had no shade trees to begin with), the weeds began growing rapidly following the hurricane. Dividing the weed community into low lying grasses, sedges, and other non-scandent vegetation versus vines and lianas, the first category began its invasion immediately after the hurricane. Those farmers who were healthy enough to clear the weeds or had a strong family or community support, had resources to hire labor to do so, or used herbicides (which are expensive), rendered the farm resilient through their actions. A study of Jamaican farmers after hurricane Dean in 2007 also highlight the importance of personal resourcefulness and ingenuity, as well as strong community support for the coping capacity in the aftermath of hurricanes^48^. Those farmers who were poorer and/or infirm, or had no family or community support, had no possibility to control weeds early on. Once the vines made it above the lower weed community and began climbing the coffee trees, all was lost (Fig. 4b).

Although this conclusion is based on data from only 10 farms and should be taken with caution, it was qualitatively corroborated by our visit to many other farms in the region that show complete vine coverage. It is evident through direct observation and casual conversations with coffee farmers that vines are of overwhelming importance in Puerto Rico, perhaps because it is an island. To our knowledge the comparison of island versus mainland vine dynamics has never been treated in the literature, but the overwhelming importance of vines in Puerto Rico in comparison to mainland areas is easily observable (our collective experience includes observations on coffee farms in Mexico, Colombia, Nicaragua, Costa Rica, Guatemala, and Brazil). Perhaps because of the large number of non-native vines in Puerto Rico (there are 386 species of vines in Puerto Rico, 112 of which are non-native^51^), the natural enemies that tend to keep them in check in the mainland are not present in the island and their proliferation go uncontrolled. Nowhere is this more important than in agriculture generally, but more importantly in coffee production. Once the vines cover the coffee plants, the use of herbicides is not possible and the labor required to control them is dramatically intensified since when tendrils are tightly wrapped around this perennial crop they cannot simply be cut. They must be “unwound” from the branches they occupy (personal experience) and care must be taken to avoid severe damage to the coffee plant. It is akin to a critical transition once the vines take over. These processes make the resilience of coffee farms to hurricane disturbance in Puerto Rico partly a function of canopy cover and other direct management factors, but also, and perhaps more importantly, a function of the socioeconomic position of the farmer. Those with the ability to control weeds immediately after the hurricane were those with sufficient capital, whether social such as family members, or monetary such as funds to purchase herbicides or pay for labor. In other words, the resilience of the system, viz-a-viz weeds (of which vines are the most important), is a property of socioecological factors.

## Methods

**Figure 8:**
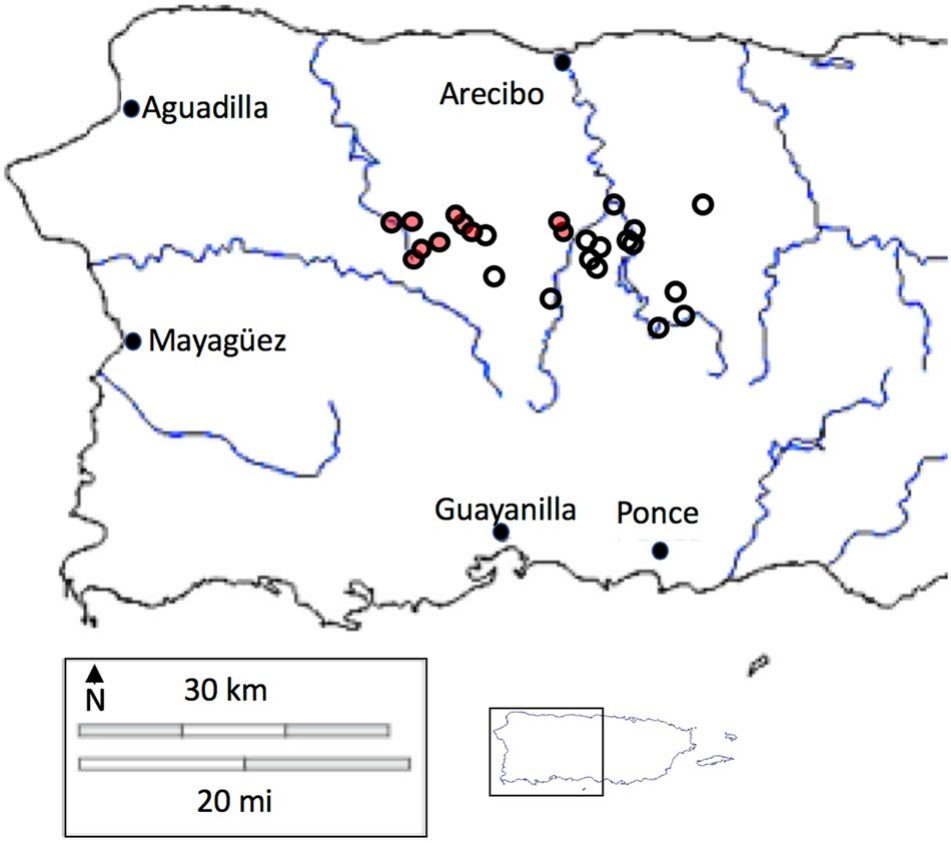
Position of sampled farms – basic vegetation surveys on all farms, versus intensive sampling of key pest species on the farms indicated by the red shaded points. Map constructed from Google Earth image by authors.

A survey of 36 farms was conducted in 2013 wherein canopy cover and other variables were measured between March and August, 2013^52^. Of those 36 farms, 8 have been completely abandoned after the hurricane and the remaining 28 were resurveyed (between Feb 9, 2018 and July 25, 2018; For information on farm location, elevation, slope and aspect see Table S1 in Supplementary Materials on line), less than one year after hurricane Maria hit the island in September 2017 (Fig. 8). In both cases, a small quadrat was established in a part of each of the farms, choosing the site to be representative of the farm as a whole. Quadrats were not in the same sites in 2013 and 2018, but in both cases were chosen with the intent of representing the general nature of the farm. Canopy cover was estimated from 9 equidistant points in a 25 × 25 m plot using a concave densiometer (Forestry Suppliers, Jackson, Mississippi, USA) in 2013, and from 5 equidistant points in a 10 × 10 m plot in 2018, measured with the digital densiometer app “CanopyApp” (University of New Hampshire ©). The average is reported. Farmer interviews conducted in 2013 provided data on expenditures on pesticides, herbicides, and fertilizers. Informed consent was obtained from each farmer interviewed. Interviews were carried out in accordance with the University of Michigan Institutional Review Board and was exempt from IRB approval (HUM00077411).

A subset of ten of the 28 farms (Fig. 8) was chosen to do a detailed examination of several key pests in the system, in an attempt to explore initial evidence of resilience in the context of pests and their potential control as a function of management intensity. Farms were chosen based on accessibility after the hurricane. The key pests were, 1) vines (many species were encountered, but all lumped into the category “vines,”), 2) coffee berry borer (*Hypothenemus hampeii*), 3) flatid leafhopper (*Petrusis epilepsis*), 4) coffee leaf miner (*Leucoptera coffeella*), 5) helmet scale, (*Saissetia coffeae*), 6) green coffee scale (*Coccus viridis*), and 6) coffee rust disease (*Hemileia vastatrix*). Observations were made on a 100 m transect on each of the ten farms (transect positions were selected so as to represent the overall management style of the farm), on coffee plants spaced approximately 2 meters from one another.

Flatid leafhoppers were measured by counting all observed leafhoppers on a coffee plant during a three-minute observation period. Leaf miners were estimated by recording the number of miners on each leaf of ten haphazardly chosen branches (covering the low, medium and upper part of the plant). Coffee berry borers were measured by counting the number of bored berries in 100 berries in three branches (top medium and low) and calculating the proportion of damaged berries. Helmet scales, green coffee scales, and green coffee scales attacked by *L. lecanii* were placed in one of four categories, 0 = none observed, 1 = few observed (<100), 2 = between 100 and 400, and 3 =  > 400. Coffee rust was assessed by counting all leaves attacked by the rust. Vine coverage was assesed by estimating the percent of the plant covered with vines. This assessment was performed by three observers independently and the average was recorded.

Interviews with farmers in the 10 intensively sampled farms were conducted in August 2018 in order to gather data on farmer’s response to the hurricane. Interviews were carried out in accordance with the University of Michigan Institutional Review Board (IRB approval HUM00137050). Interview questions are supplied in the supplementary material.

## Supplementary information

Supplementary Information

## Data Availability

The datasets generated during and/or analyzed during the current study are available from the corresponding author on reasonable request.
