# Supplementary material for: Response of Coffee Farms to Hurricane Maria: Resistance and Resilience from an Extreme Climatic Event
Source: Sci Rep. 2019 Oct 30;9:15668. doi: 10.1038/s41598-019-51416-1 (PMC6821701; doi:10.1038/s41598-019-51416-1)
Supplement: Supplementary file 1 — Supplementary Information [file 41598_2019_51416_MOESM1_ESM.docx]

Supplementary on line materials

**RESPONSE OF COFFEE FARMS TO HURRICANE MARIA: RESISTANCE AND RESILIENCE FROM AN EXTREME CLIMATIC EVENT**

Ivette Perfecto^a^, Zachary Hajian-Forooshani^b^, Aaron Iverson^c^, Amarilys D. Irizarry^a^, Javier Lugo-Perez^d^, Nicholas Medina^b^, Chatura Vaidya^b^, Alexa White^b^, John Vandermeer^b^

^a^ School for Environment and Sustainability, University of Michigan, Ann Arbor, MI.

^b^ Department of Ecology and Evolutionary Biology, University of Michigan, Ann Arbor, MI.

^c^ Department of Ecology and Evolutionary Biology, Cornell University, Ithaca, NY.

^d^ Departamento de Biología, Universidad de Puerto Rico, Recinto de Utuado, Utuado, PR.

*Corresponding Author: Ivette Perfecto, tel: +1 (734) 604-4331; Email: perfecto@umich.edu


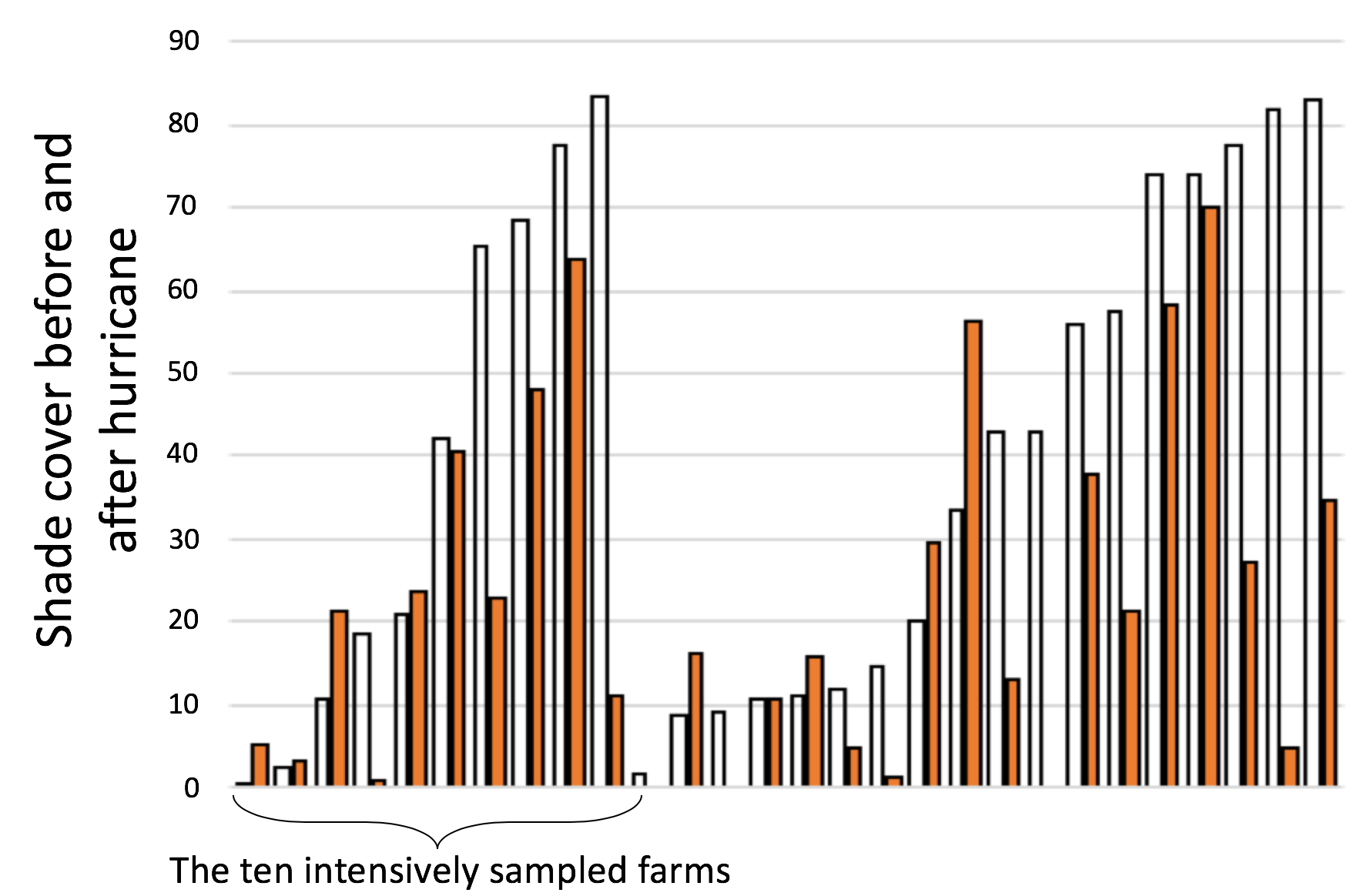


*Figure S1. Bar graph presentation of same data, open bars before the hurricane, shaded bars after the hurricane – ordered first with the intensively sampled farms versus the rest and according to pre-hurricane canopy cover within those categories.*

Table S1. Location, elevation, slope and aspect for 28 farms used in the study.

| Region/Number of the farm within region | Elevation | Latitude | Longitude | Slope | Aspect |
| --- | --- | --- | --- | --- | --- |
| UTUADO 23 | 251.51 | 18.2633 | -66.6485 | 29.32 | 48.16 |
| UTUADO 26 | 263.04 | 18.2387 | -66.6385 | 9.98 | 255.66 |
| UTUADO 22 | 284.67 | 18.2467 | -66.6449 | 5.79 | 16.29 |
| UTUADO 35 | 375.00 | 18.2806 | -66.6740 | 6.39 | 33.21 |
| UTUADO 3 | 378.88 | 18.2879 | -66.7703 | 8.12 | 259.08 |
| UTUADO 34 | 382.01 | 18.2973 | -66.6237 | 6.23 | 293.32 |
| UTUADO 21 | 393.30 | 18.2309 | -66.7337 | 12.23 | 106.58 |
| CIALES 1 | 403.64 | 18.2976 | -66.5413 | 10.96 | 229.40 |
| UTUADO 20 | 417.82 | 18.2796 | -66.8284 | 11.85 | 320.73 |
| UTUADO 24 | 425.03 | 18.2569 | -66.6359 | 24.17 | 295.26 |
| UTUADO 31 | 430.76 | 18.2811 | -66.8095 | 19.27 | 149.79 |
| UTUADO 19 | 453.86 | 18.2774 | -66.7620 | 14.85 | 20.38 |
| UTUADO 17 | 472.13 | 18.2692 | -66.7419 | 18.68 | 161.06 |
| UTUADO 28 | 481.26 | 18.2631 | -66.7844 | 18.70 | 185.46 |
| UTUADO 16 | 483.95 | 18.2718 | -66.6703 | 16.20 | 135.55 |
| UTUAD0 25 | 518.31 | 18.2099 | -66.6817 | 11.39 | 129.83 |
| UTUADO 18 | 524.28 | 18.2724 | -66.7543 | 22.13 | 116.88 |
| JAYUYA 2 | 526.44 | 18.2161 | -66.5672 | 16.82 | 187.61 |
| UTUADO 33 | 529.24 | 18.1901 | -66.6789 | 6.70 | 60.01 |
| UTUADO 27 | 534.86 | 18.2731 | -66.6041 | 15.40 | 84.19 |
| UTAUDO 13 | 565.86 | 18.2562 | -66.8017 | 7.82 | 112.77 |
| UTUADO 32 | 574.51 | 18.2466 | -66.8071 | 34.93 | 168.24 |
| JAYUYA 4 | 599.02 | 18.1949 | -66.5591 | 4.27 | 279.02 |
| UTUADO 30 | 632.67 | 18.2601 | -66.6058 | 24.54 | 262.71 |
| UTUADO 10 | 659.19 | 18.2640 | -66.6110 | 22.22 | 350.37 |
| CIALES 3 | 688.01 | 18.2551 | -66.5344 | 13.38 | 333.62 |
| UTUADO 29 | 735.03 | 18.2488 | -66.6088 | 6.77 | 339.08 |
| JAYUYA 1 | 855.93 | 18.1836 | -66.5828 | 10.49 | 323.36 |

*Interview Questions*

1. **Farm general information**:

Area of the farm (has.): _________________Area cultivated in coffee (has.): _______________________
Area in forest (has.): __________________ Comments: ___________________________________
Area cultivated in arabica coffee: __________________ robusta coffee: _______________
Other crops (in addition to coffeee): ___plantain ___bananas ___citrus fruits ___root crops: _________________
How much goes to the market? ___________________________________________________
Type of management: ___unshaded ___shaded ___both: If both, what proportion of the coffee is allocated to each type of management? ___________________________________________________________________
Only shaded coffee: in general, what tree species do you have on your farm?
_____________________________________________________________________________________________
Other trees (e.g. fruit trees): _________________________________________________________________________
On average, what has been the production of the farm for the last 5 years (in *quintales*): ____________________________________
What are the main pest problems on the farm?, ___weeds (including vines) ___insects ___ diseases (for example, coffee rust)?
Use of: ___insecticides ___herbidices ___fungicides ___fertilizer ___no use of agrochemichals
Do you think it is important to reduce the use of agrochemicals? _______________________________________________

1. **Information about the management of the farm after the hurricane**:

Impact: What kind of damage did your farm experienced? ___How much coffee production was lost? ___estimate number or percentage of coffee plants destroyed, ___felled ___How many trees fell or were severely damaged / other damage:__________________________________________________________
How many coffee berries were lost? ______________________________________________________________
coffee plants? ______________________________________________________________________________
Other crops? _______________________________________________________________________________
Fruit trees? _____________________________________________________________________________ Recovery: How much time after the hurricane did you waited before you were able to work on the farm ? ___________________________________
What resources did you used? (e.g.. machinery, machete, trimmer?)______________________________________________
Did you hired workers? ___No ___Yes / How many? ______________________________________________
Did you received any help from ___the government ___neighbors ___family ____solidarity brigades Other:______________________________
What type of help have you received to recover your losses and restore yourfarm? (loans, Ffderal programs, national programs) _________________________
Was your coffee insured (2017)? ___Yes ___No / When did you received a payment from the insurance? __________________________
Provide an estimate for your production for 2018? _____________________________________________
What has been the most difficult aspect of the farm recovery process? __________________________________________
How do you see the future of your coffee farm?
